# Emamectin·chlorfenapyr-induced fatal leukoencephalomyelopathy with delayed hyperthermia: insecticide endanger public safety

**DOI:** 10.3389/fneur.2024.1449728

**Published:** 2024-12-06

**Authors:** Xun Li, Yun Yang, Yajing Zhang, Xuebin Zhang, Na Zhao, Wei Yue

**Affiliations:** ^1^Department of Neurology, Tianjin Huanhu Hospital, Tianjin, China; ^2^Tianjin Huanhu Hospital, Tianjin, China

**Keywords:** emamectin·chlorfenapyr, leukoencephalomyelopathy, hyperthermia, lymphocyte, fatal outcome

## Abstract

**Background:**

Emamectin·chlorfenapyr is a compound comprising chlorfenapyr and emamectin benzoate that is widely used in agriculture. Chlorfenapyr toxicity has been verified in animals; however, its true mechanism and progression in humans remain to be elucidated. Cases of emamectin·chlorfenapyr poisoning are seldom.

**Case presentation:**

We present a case of a 65-year-old female who attempted suicide by consuming 30 g of 9.5% chlorfenapyr and 0.5% emamectin benzoate 14 days before admission to our hospital. Laboratory tests revealed extremely high creatinine kinase levels upon admission. Magnetic resonance imaging revealed diffuse and symmetric T2 hyperintensities in the entire white matter tract of the brain and spinal cord, and cytological smears of the cerebrospinal fluid showed abnormal lymphocyte aggregation. The patient died 19.5 h after admission owing to cardiopulmonary arrest and hyperthermia.

**Conclusion:**

Further research is needed on how to perform flow cytometry in patients with emamectin·chlorfenapyr intoxication, and to elucidate the immunological mechanism underlying the inflammatory response caused by emamectin·chlorfenapyr and provide new insights into antidote development.

## Introduction

Emamectin·chlorfenapyr is a formulation that contains 9.5% chlorfenapyr and 0.5% emamectin benzoate (EB). EB (C104H154N2O28) is employed extensively as a broad-spectrum insecticide and pesticide in agriculture (1). It serves as a GABA receptor agonist and modifies the permeability of chloride ions in membranes (2). Chlorfenapyr (C15H11BrClF3N2O), a pyrrole insecticide, is widely used in the cultivation of vegetables and fruits (3). It inhibits the normal oxidative phosphorylation in mitochondria, leading to a decrease in adenosine triphosphate (ATP) and impairing the function of organs that require oxygen (4).

Agriculture serves as a crucial industry pillar in the Asia-Pacific region. The prevalent use of pesticides enhances the accessibility of these toxic substances, which may be ingested either accidentally or intentionally in suicide attempts. Initial clinical symptoms of poisoning are often not immediately apparent. Approximately 7 days post-exposure, toxicity can manifest with delayed onset. Both EB and chlorfenapyr are associated with depression of the nervous system (2). Additionally, common manifestations of chlorfenapyr poisoning include hyperthermia and rhabdomyolysis (5).

Chlorfenapyr exposure, though not necessarily lethal, has resulted in significant mortality in the Asia-Pacific region (4–6) and the United States (7). While the toxicity of chlorfenapyr has been established in animal studies, its exact mechanism and progression in humans remain elusive. This paper describes a fatal incident involving a 65-year-old female who ingested a bottle of emamectin·chlorfenapyr in an attempt to commit suicide. Unlike previous reports on chlorfenapyr and EB poisoning, this case study includes results from cerebrospinal fluid tests and cytological smears.

## Case report

A 65-year-old female patient was admitted to our emergency department presenting with urinary incontinence and a 5-day history of progressive weakness and hypoesthesia in the lower extremities. She had a medical history of hypertension, type 2 diabetes mellitus, and depression. 14 days prior to admission, the patient had attempted suicide by ingesting 30 g of 9.5% chlorfenapyr and 0.5% EB.

Electrocardiogram monitoring indicated the following vital signs: body temperature at 37.6°C, heart rate at 73 beats per minute, respiratory rate at 22 breaths per minute, and blood pressure at 140/69 mmHg. Neurological examination revealed motor weakness in both legs (grade 0/grade 0) and diminished response to acupuncture pain below the T8 dermatome. The patient was fully conscious with a Glasgow Coma Scale (GCS) score of E4, V5, M6.

Toxicity screening revealed trace amounts of chlorfenapyr and emamectin benzoate; cholinesterase inhibitors, illicit drugs, and benzodiazepines were not detected. Prior computed tomography of the chest and abdomen conducted in our emergency room showed no significant abnormalities. Laboratory results indicated a markedly elevated creatine kinase level of 2,406 IU/L (normal range: 40–200 IU/L) and mild liver dysfunction, evidenced by an aspartate aminotransferase level of 85 IU/L (normal range: 13–35 IU/L) and an alanine transaminase level of 47 IU/L (normal range: 7–40 IU/L). Routine blood tests disclosed a white blood cell count of 9.93*10^9/L (normal range: 3.5–9.5*10^9/L) and a neutrophil percentage (NEU%) of 82.7% (normal range: 40–75%). The procalcitonin level was slightly abnormal at 0.081 ng/mL (normal range: 0–0.046 ng/mL).

**Figure 1:**
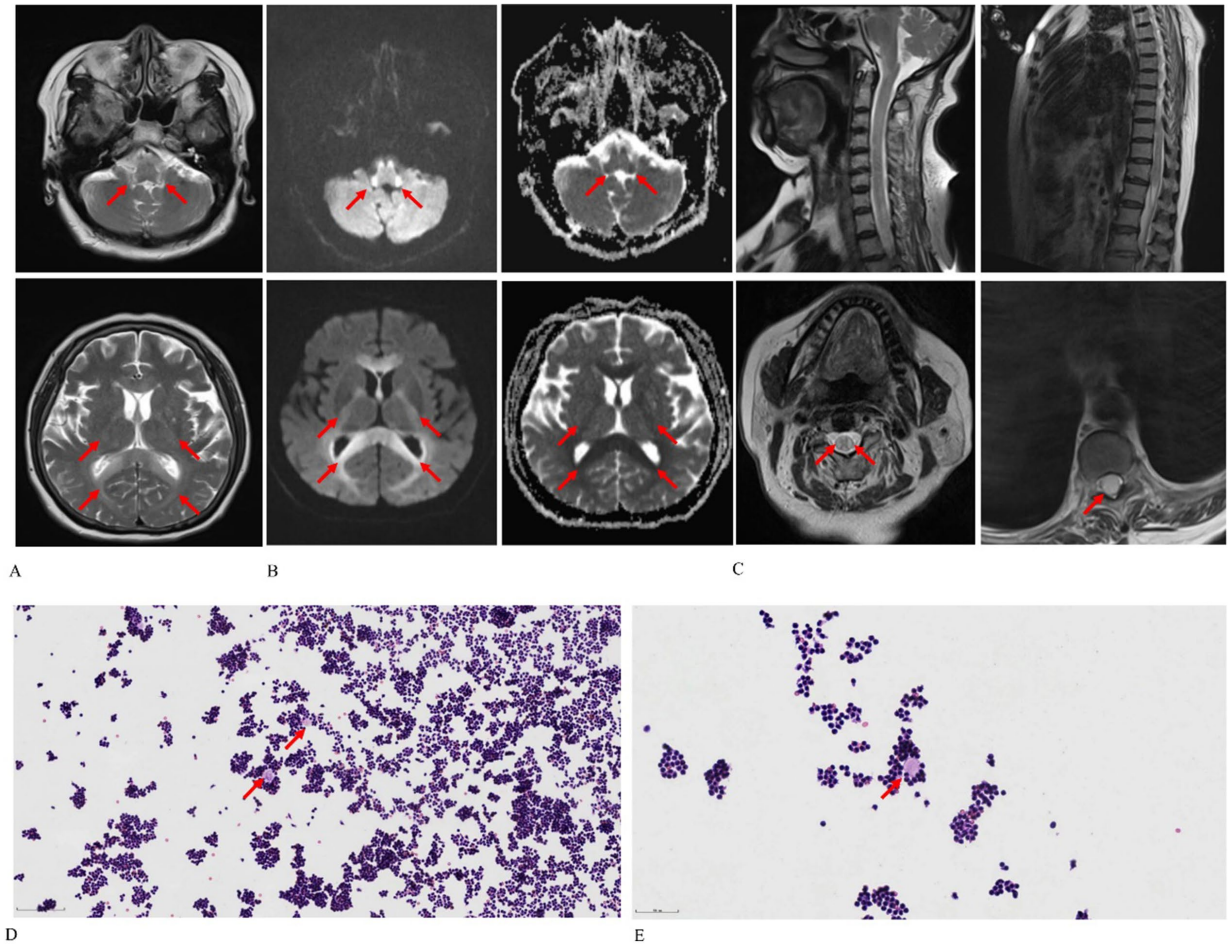
Chlorfenapyr and emamectin benzoate-induced leukoencephalomyelopathy in a 65-year-old female patient. **(A)** Axial T2-weighted images reveal diffuse and bilaterally symmetrical leukoencephalopathy affecting the dentate nucleus of the cerebellum, ventral medulla, bilateral inferior cerebellar peduncles, pons, midbrain, bilateral cerebral peduncles, bilateral corticospinal tracts, corpus callosum, and bilateral parieto-occipital white matter. **(B)** Axial diffusion-weighted imaging and apparent diffusion coefficient maps demonstrate cytotoxic edema and reduced Brownian motion. **(C)** Sagittal T2-weighted spine images display swelling and hyperintensity throughout the spinal cord, particularly in the cervical region and conus medullaris. **(D,E)** Cytological smears of cerebrospinal fluid [optical microscope: Hematoxylin–eosin stain, 100× **(D)**, 200× **(E)**] show lymphocytes clustering around protein-like material in a wreath-like formation, as indicated by the red arrow.

Magnetic resonance imaging (MRI) of the patient’s brain and spine was performed using a 3.0 T unit (MAGNETOM Skyra; Siemens Healthcare GmbH, Erlangen, Germany). The brain MRI exhibited diffuse, bilaterally symmetrical leukoencephalopathy affecting the dentate nucleus of the cerebellum, ventral medulla, bilateral inferior cerebellar peduncles, pons, midbrain, bilateral cerebral peduncles, bilateral corticospinal tracts, corpus callosum, and bilateral parieto-occipital white matter (Figure 1). T2-weighted imaging (T2WI) showed lesions with increased signal intensity (Figure 1A). Diffusion-weighted imaging and an apparent diffusion coefficient map indicated cytotoxic edema and diminished Brownian movement (Figure 1B). The spinal cord was characterized by swollen, hyperintense lesions on T2WI, particularly noted in the cervical spinal cord and conus medullaris (Figure 1C). A lumbar puncture conducted in the emergency room revealed that the cerebrospinal fluid pressure was 240 mmH2O, and the fluid appeared as a yellowish, cloudy liquid containing flocculent material. The total cell count in the cerebrospinal fluid was 6000*10^6/L, with a white blood cell count of 5200*10^6/L and NEU% at 94%. Cerebrospinal fluid analysis yielded the following results: chloride at 114 mmol/L (normal range: 120–132 mmol/L); glucose at 2.37 mmol/L (normal range: 2.5–4.5 mmol/L); lactate at 7.7 mmol/L (normal range: 0.6–2.2 mmol/L); and protein at 1.57 g/L (normal range: 0.15–0.45 g/L). Next-generation sequencing of the cerebrospinal fluid detected no pathogens. Tests for autoimmune encephalitis-related antibodies, demyelinating disease-related antibodies, paraneoplastic nerve syndrome antibodies, cerebrospinal fluid oligoclonal bands, and serum oligoclonal bands were negative. A cytological smear of the cerebrospinal fluid unveiled some lymphocytes clustered around proteinaceous material in a wreath-like configuration (Figures 1D,E).

Based on the imaging and laboratory findings, toxic leukoencephalomyelopathy was suspected. The patient’s condition deteriorated on the second day, and hyperthermia ensued 16 h post-admission, with body temperatures rising to 39–40°C. Antipyretic medications proved ineffective. The Glasgow Coma Scale (GCS) was assessed at E3, V3, M5, indicating a progressive deterioration in consciousness. 18 h after admission, the patient developed tachycardia (heart rate of 110 beats/min) and tachypnea (respiratory rate of 30 breaths/min) with shallow breathing. Consequently, steroid pulse therapy was initiated. The patient’s percutaneous oxygen saturation fluctuated between 80 and 90%, and a Venturi mask delivering 10 L/min was employed to enhance oxygenation. 19 h post-admission, arterial blood gas analysis revealed hypoxemia (pH 7.31; partial pressure of CO2 50 mmHg; partial pressure of O2 44 mmHg; HCO3 26.5 mmol/L). The patient then entered a deep coma, reflected by a GCS score of E1, V1, M1. Immediate transfer to the intensive care unit was arranged, where tracheal intubation was performed. Unfortunately, the patient succumbed to cardiopulmonary arrest 19.5 h after admission, following the family’s issuance of do-not-resuscitate orders.

## Discussion

Reports on the full spectrum of manifestations of emamectin-chlorfenapyr poisoning in humans are scarce. A review of the literature was conducted to identify characteristics of such poisoning, yielding a total of 17 reported cases of emamectin-chlorfenapyr poisoning. The details are presented in Table 1. Among these cases, the mean age of the patients was 46.11 ± 15.82 years, and the average hyperthermal temperature was 40.08 ± 1.46°C. Only three cases in which the patient survived have been documented, resulting in a mortality rate of 84.21%. More than half of the patients died in hospital within 1 day of admission.

**Table 1:** List of reports documenting toxicity due to emamectin·chlorfenapyr.

Reference	Year	Country	Age (years)	Sex	Route	Amount of poisoning	Highest BT (°C)	AMS	Elevated CK (IU/L)	AKI	Management	LOS (days)	Outcome
(4)	2010	Korea	55	Male	Oral	250 mL of 10% chlorfenapyr	40.9	Yes	Yes (10507) Yes		Gastric lavage and activated charcoal, and intubation	5	Mortality
(18)	2012	Korea	49	Male	Oral	200 mL	40	Yes	Yes (14336)	Unknown	Intubation and mechanical ventilation	7	Mortality
(9)	2013	India	28	Female	Oral	Unknown	Unknown	Yes	Unknown	Unknown	Gastric lavage and supportive treatment, intubation	1	Mortality
(19)	2013	Korea	74	Male	Intra-abdominal injection	20 mL	Unknown	Unknown	Unknown	Unknown	Emergency surgery and fluid drainage	Unknown	Mortality
(10)	2014	Korea	41	Female	Oral	20 mL	40.7	Yes	Yes (3081)	No	Intravenous fluid hydration and urine alkalinization, intubation and mechanical ventilation	0.83	Mortaliy
(11)	2015	Korea	61	Female	Oral	10 mL	38.3	Unknown	Yes (859)	Unknown	Gastric lavage and activated charcoal	19	Survival
(6)	2016	Korea	44	Female	Oral	Unknown	Unknown	Unknown	Unknown	Unknown	Steroid pulse therapy	Unknown	Survival
(7)	2017	United States	42	Male	Oral	Approximately 300 mL of 21% chlorfenapyr and 500 mL vodka	Unknown	Yes	Unknown	Unknown	Gastric lavage and activated charcoal	Unknown	Mortality
(20)	2018	Korea	44	Female	Oral	10-20 mL	Unknown	Unknown	Unknown	Unknown	Gastric lavage and high-dose intravenous corticosteroid pulse therapy	Unknown	Survival
(21)	2019	Korea	49	Male	Dermal	Unknown	41.5	Yes	Yes (4484)	Unknown	Intubation, sedation and analgesia, intravenous administration of paracetamol and tepid sponging	0.46	Mortality
(22)	2020	China	66	Male	Oral	20 mL	38.5	Yes	Yes (8174)	Yes	Gastric lavage and supportive treatment, intubation	5	Mortality
(12)	2021	China	50	Female	Oral	60 mL	40	Yes	Yes (1960)	Unknown	Intravenous fluid hydration and urine alkalinization, intubation and mechanical ventilation	<1	Mortality
(12)	2021	China	50	Male	Transnasal	Unknown	41	Yes	Yes (1590)	Unknown	Intravenous fluid hydration and urine alkalinization, intubation and mechanical ventilation	<1	Mortality
(12)	2021	China	38	Male	Transnasal	Unknown	40	Yes	Yes (5945)	Unknown	Intravenous fluid hydration and urine alkalinization, intubation and mechanical ventilation	<1	Mortality
(12)	2021	China	32	Female	Oral	5 mL	41.8	Yes	Yes (3762)	Unknown	Intravenous fluid hydration and urine alkalinization, intubation and mechanical ventilation	1	Mortality
(8)	2022	China	21	Male	Oral	Unknown	39	Yes	Yes (933.74)	No	Intravenous fluid hydration and urine alkalinization, intubation and mechanical ventilation	1	Mortality
(8)	2022	China	55	Male	Oral	60 mL	37.3	Yes	Yes (820.48)	No	Emergency surgery and fluid drainage, intubation	<1	Mortality

CK, creatinine kinase; normal range, 40–200 IU/L; LOS, length of stay; BT, body temperature; AMS, altered mental status; AKI, acute kidney injury.

Mammalian species exhibit low sensitivity to EB due to the chemical’s minimal affinity for gamma-aminobutyric acid and the relative impermeability of the blood–brain barrier (2). Chlorfenapyr, classified as moderately hazardous, disrupts the conversion of adenosine diphosphate to ATP by targeting mitochondria, thereby halting ATP production and leading to energy depletion, cellular dysfunction, and death (5). Emamectin·chlorfenapyr, a pesticide combination, is formulated to delay resistance development in insect pests and enhance synergistic effects. EB synergistically contributes to oxidative phosphorylation decoupling induced by chlorfenapyr, leading to cell death (8). This mechanism likely underlies the predominant features of emamectin·chlorfenapyr toxicity, which include decreased consciousness, respiratory failure, rhabdomyolysis, and demyelination of the brain and spinal cord, targeting organs with high energy demands.

In previous studies on chlorfenapyr poisoning, the clinical trajectory typically exhibits a latent period of up to 14 days, succeeded by a rapid progression to death (4, 9, 10). Kang et al. (10) postulated that the incubation period for chlorfenapyr toxicity correlates with the time needed for the pro-insecticide to convert into the active toxicant. Similarly, our patient underwent a latent phase of approximately 9 days, during which she displayed no discernible symptoms until the onset of paraplegia. Furthermore, the exact lethal dosage of chlorfenapyr or emamectin·chlorfenapyr remains undetermined, owing to variations in medical settings and individual biochemical characteristics. In a documented survival case (11) following chlorfenapyr ingestion, the patient, who ingested about 10 mL, did not show hyperintensity in the central nervous system and received immediate treatment through early gastrointestinal decontamination, including gastric lavage and whole-bowel irrigation. Conversely, our patient experienced a concealed progression of the condition, with severe damage to her brain and spinal cord upon hospital admission, thus missing the critical window for effective treatment. EB may also contribute synergistically to the lethality of this process.

Other typical clinical manifestations of chlorfenapyr poisoning observed in our patient include rhabdomyolysis and hyperthermia. Delayed hyperthermia, a common observation in cases of chlorfenapyr intoxication, was also noted. In a case report from China, similar to ours, tracheal intubation appeared to hasten clinical decline (12), This deterioration may be associated with pulmonary infections and respiratory muscle failure following tracheal intubation, although the exact mechanisms are yet to be elucidated. Rhabdomyolysis and hyperthermia are also indicative of malignant hyperthermia, a rare condition triggered by certain anesthetics. In this patient, no anesthetics or medications known to induce malignant hyperthermia were administered. It is hypothesized that the lipophilic nature of chlorfenapyr leads to its accumulation in striated muscles, uncoupling oxidative phosphorylation in mitochondria, disrupting ATP synthesis, and inducing cell death, which elevates creatine kinase levels. Consequently, this triggers non-shivering thermogenesis in brown adipose tissue, culminating in hyperthermia (10).

Few cases of chlorfenapyr intoxication in humans that include integrated radiological data have been reported. Tharaknath et al. (9) reported a case of chlorfenapyr poisoning with diffuse and bilateral hyperintensity in the white matter of the brain and spinal cord on T2WI MRI; their case was very similar to ours. Baek et al. (6) reported a case of survival after chlorfenapyr intoxication and found reversible signal changes in the white matter tracts throughout the brain, brainstem, and spinal cord after 75 days of follow-up visits. Other similar radiological features reported by Kang et al. (10) also indicated a low white matter density in the whole brain tissue on computed tomography scans. In a previous animal experiment involving rats with chlorfenapyr poisoning, vacuolar myelopathy and mild myelin sheath swelling were found in a variety of structures ranging from the subcortical areas to the brainstem and spinal cord in neurohistopathological examinations (13); this is consistent with MRI findings in clinical case reports. However, the pathophysiology of the susceptibility of the white matter tracts alone is unknown. We presume that chlorfenapyr tends to invade and accumulate in the myelin sheath owing to its weak lipophilic acid features, and causes myelin disintegration, the presence of which is shown on MRI. In addition, heat-regulating centers in the hypothalamus are believed to be involved in chlorfenapyr cases, causing hyperthermia (13).

A detailed interpretation of why the cerebrospinal fluid had a count of a white blood cell count of 5200*10^6/L and NEU% of 94% could be assumed that toxicant destroyed blood–brain barrier and raised immunologic derangement, which recruiting inflammatory cells. Decidedly few case reports that present cerebrospinal fluid consequences have been published. Baek et al. (6) reported that the cerebrospinal fluid test results of a patient with diffuse abnormal signals in the brain and spinal cord were normal. However, using next-generation sequencing and cytometric bead array, we confirmed that the cerebrospinal fluid of our patient showed a secondary inflammatory process with no pathogenic microorganisms or related antibodies. In the cytological smear of the cerebrospinal fluid, we observed that some lymphocytes surrounded the protein-like material and lined up in a wreath-like pattern. We speculated that the protein-like material may be a type of myelin tissue that has been shed and that contains abundant tralopyril, which is a metabolized form from chlorfenapyr (14). In addition, chlorfenapyr could activate some lymphocyte subsets, which, in turn, could gather around the exogenous substance. Only one case report of methadone intoxication may provide some evidential support of our speculations. Repple et al. (15) performed flow cytometry on the cerebrospinal fluid of methadone-intoxicated patients and found cell composition alterations that were characterized by the transformation of monocytes from the classical (CD14^+^CD16^−^) to the nonclassical (CD14^+^CD16^+^) and a switch from CD56^bright^ nonkilling cells to CD56^dim^ nonkilling cells. Notably, CD14^+^CD16^+^ monocytes and CD56^dim^ nonkilling cells are pro-inflammatory cells that may be involved in delayed encephalopathy (16). These findings from Repple et al. may explain the lymphocyte aggregation observed in the current case, which provides the first reported cerebrospinal fluid findings of chlorfenapyr intoxication.

Pesticide poisoning is a significant global public health issue, resulting in approximately 110,000–168,000 deaths annually, predominantly in Asia. The World Health Organization (WHO) reported that accidental poisonings led to around 350,000 deaths in 2000. A further estimate indicates that in 2012, unintentional poisoning claimed the lives of 193,000 people globally, with 84 percent of these fatalities occurring in low- and middle-income countries (17). Developing nations experience higher rates of suicide via poisoning, notably through pesticides. Conversely, in high-income countries, substances used in suicide attempts include psychotropic drugs, painkillers, antihistamines, antidepressants, psychoactive medications, and sedative hypnotics. It raises concerns that suicide remains an increasingly prevalent “illness.” Pesticide poisoning represents merely one method of suicide; thus, focusing on prevention is substantially more critical than merely addressing the effects. Health systems must continue to provide special attention to the mental health of their citizens.

Limitations in this case reports include that: (1) The precise dosage of emamectin·chlorfenapyr is still unclear as described with 30 g. The reason goes that the pesticide were produced by unqualified manufacturer in Chinese mainland, and the manufacturer did not have production permit, so we could not know the specific dosage form. (2) Chlorfenapyr is metabolized to tralopyril in the body and persists for a prolonged period, but the blood concentration levels observed for tralopyril is unreviewable for the interpretation that blood concentration levels were very low at the time when testing, there is no idea about the accurate information. Although there are deficiencies on clinical observations, the diagnosis is still can be done by comprehensive assessment.

## Conclusion

We present a fatal case of leukoencephalomyelopathy induced by emamectin·chlorfenapyr with delayed hyperthermia and rapid deterioration. The latent period from emamectin·chlorfenapyr exposure to paraplegia onset was less than 9 days, and MRI revealed bilateral, symmetric, and diffuse T2 hyperintensity of the entire white matter tract in the brain and spinal cord. In the cytological smear of the cerebrospinal fluid, we observed that some of the lymphocytes gathered in a wreath-like pattern surrounding a protein-like material; this is the first report of cerebrospinal fluid microscopic characteristics in emamectin·chlorfenapyr intoxication. Further research is needed to clarify how to perform flow cytometry on patients with emamectin·chlorfenapyr intoxication, to elucidate the immunological mechanism underlying the inflammatory process caused by emamectin·chlorfenapyr, and to provide new insights into antidote development.
